# Methods to Determine the Transcriptomes of Trypanosomes in Mixtures with Mammalian Cells: The Effects of Parasite Purification and Selective cDNA Amplification

**DOI:** 10.1371/journal.pntd.0002806

**Published:** 2014-04-17

**Authors:** Julius Mulindwa, Abeer Fadda, Clementine Merce, Enoch Matovu, John Enyaru, Christine Clayton

**Affiliations:** 1 Zentrum für Molekulare Biologie der Universität Heidelberg (ZMBH), DKFZ-ZMBH Alliance, Heidelberg, Germany; 2 College of Veterinary Medicine, Animal Resources & Biosecurity, Makerere University, Kampala, Uganda; 3 College of Natural Sciences, Makerere University, Kampala, Uganda; New York University School of Medicine, United States of America

## Abstract

Patterns of gene expression in cultured *Trypanosoma brucei* bloodstream and procyclic forms have been extensively characterized, and some comparisons have been made with trypanosomes grown to high parasitaemias in laboratory rodents. We do not know, however, to what extent these transcriptomes resemble those in infected Tsetse flies - or in humans or cattle, where parasitaemias are substantially lower. For clinical and field samples it is difficult to characterize parasite gene expression because of the large excess of host cell RNA. We have here examined two potential solutions to this problem for bloodstream form trypanosomes, assaying transcriptomes by high throughput cDNA sequencing (RNASeq). We first purified the parasites from blood of infected rats. We found that a red blood cell lysis procedure affected the transcriptome substantially more than purification using a DEAE cellulose column, but that too introduced significant distortions and variability. As an alternative, we specifically amplified parasite sequences from a mixture containing a 1000-fold excess of human RNA. We first purified polyadenylated RNA, then made trypanosome-specific cDNA by priming with a spliced leader primer. Finally, the cDNA was amplified using nested primers. The amplification procedure was able to produce samples in which 20% of sequence reads mapped to the trypanosome genome. Synthesis of the second cDNA strand with a spliced leader primer, followed by amplification, is sufficiently reproducible to allow comparison of different samples so long as they are all treated in the same way. However, SL priming distorted the abundances of the cDNA products and definitely cannot be used, by itself, to measure absolute mRNA levels. The amplification method might be suitable for clinical samples with low parasitaemias, and could also be adapted for other Kinetoplastids and to samples from infected vectors.

## Author Summary

Most experiments on African trypanosomes - including those designed to look for new drugs - have studied parasites either from culture, or from laboratory rodents. We are interested in comparing these parasites that grow in man and domestic animals, where the parasites generally have different nutrient concentrations and much lower parasitaemias than in experimental models. The most accessible way to make the comparison is to measure the amounts of mRNAs. In this paper we describe how methods that are used to purify the parasites from human cells can change the relative amounts of mRNA. We also describe a method to examine RNA from relatively small numbers of parasites that are mixed with host cells.

## Introduction

African trypanosomes live in several niches - in the blood, tissue fluids and brain of patients with second stage sleeping sickness, and in various parts of the Tsetse fly digestive tract. Nearly all experiments on African trypanosomes, however - including those designed to look for new drugs - have studied parasites either from culture, or from laboratory rodents with high parasitaemias. Drugs have to kill parasites that are living in humans or ruminants. These parasites are very difficult to study because parasitaemias are low, so we have no idea whether their metabolism is really the same as that of parasites in culture. The ideal way to assess differences between these parasites and the standard lab models would be to characterise their proteomes or metabolomes, but the numbers of parasites are insufficient. The amounts of RNA are however sufficient, for transcriptome analysis, especially since amplification techniques are available.

The most sensitive method to characterise transcriptomes is to make cDNA by reverse transcription, followed by second strand synthesis and analysis of the products by high throughput sequencing [1]. To ensure coverage that is independent of the transcript length, the mRNA is fragmented prior to cDNA synthesis [2]. To create a good library for sequencing, 50–100 ng of mRNA is generally needed. When less is available, the cDNA can be amplified. One approach is to include tags at the ends of the cDNA, and use those sequences for polymerase chain reaction amplification or linear amplification [3], [4], [5]. An alternative is to include a promoter for a bacteriophage polymerase in the cDNA primer [3], [5], [6]. Once double-stranded cDNA is made, it has the promoter at one end, and this can be used as a template for further RNA synthesis by the bacteriophage polymerase. The RNA produced in this way is once again converted to cDNA, which is then sequenced. The various amplification techniques can be qualitatively very good for transcript detection, but when tested quantitatively, they were found to cause considerable distortions in measured transcript levels [3], [5].

Several groups have obtained trypanosome transcriptomes using RNASeq [7], including quantitation of full-length developmentally regulated mRNAs [8]. Trypanosome mRNAs are very unusual because each bears, at the 5′-end, a 39 nt sequence called the spliced leader. To map the splice sites, the spliced leader sequence can be used as a primer for second strand synthesis on cDNA. This “spliced leader tagging” approach has been used both to map splice sites [9], [10], [11] and to measure changes in splice site usage in different life cycle stages [9], [10]. In addition, it has been used to assess the effects of a gene knock-out in *Trypanosoma cruzi* [12].

The study of the transcriptomes of trypanosomes from natural infections is made difficult not only by the low parasite numbers, but also by the fact that the samples contain such an excess of host cells. For all but very high parasitaemias, a purification step will be necessary in order to obtain sufficient parasite sequence reads. One option is to purify the trypanosomes away from the host cells before RNA is prepared. The alternative is to select trypanosome sequences from the RNA mixture before starting the high-throughput sequencing procedure: the spliced leader makes this possible. We here describe an optimized spliced leader priming protocol, and compare the effects of both trypanosome purification and spliced leader priming on the final transcriptome data.

## Materials and Methods

### Ethics statement

The animal work was approved by the Makerere University animal care committee, clearance number VAB/REC/13/080. Uganda has no law governing experimentation on animals, so the Makerere committee follows EU guidelines.

### Trypanosomes

To test purification methods, approximately 5000 *T. b. rhodesiense* strain Tbr729 parasites were injected into each rat. Blood was harvested at peak parasitaemia (approximately 4×10^6^ cells/ml) within 5 days post infection.

For the amplification, *Trypanosoma brucei* Lister 427 were cultured in loosely capped flat-bottomed T-flasks in an incubator at 37°C, 5% CO_2_, in a humid atmosphere. The cell densities were maintained between 2–10×10^5^ cells/ml in supplemented HMI-9 medium.

### Isolation of trypanosomes from blood

To obtain buffy coats, infected rat blood in an EDTA tube was centrifuged at 1200×g for 10 min at room temperature (25°C). (EDTA acts as an anticoagulant.) The buffy coat was pipetted off and centrifuged at 3400×g for 2 min.

For column chromatography [13] 20 ml packed slurry of DEAE cellulose resin (Sigma) pre-equilibrated in phosphate saline glucose (PSG) buffer, pH 8, was set up in a 30 ml syringe. The column was equilibrated with 3 column volumes of PSG and thereafter loaded with the anti-coagulated rat blood (approximately 5 ml) at room temperature. The trypanosomes were eluted with PSG into a 15 ml tube and then centrifuged for 10 min at 850×g at room temperature. The procedure took approximately 30–40 min and this would be similar for standard 5 ml samples of human blood.

Trypanosomes in infected rat blood were also purified by hemolysis followed by centrifugation. Briefly, to one volume of infected rat blood was added 3 volumes of Erythrocyte lysis buffer (Qiagen), then the two were gently mixed and incubated at room temperature (25°C) for 7 min before centrifugation at 850×g for 10 min (total duration approximately 20 min). The content of the Qiagen solution is not known but most common procedures involve the use of isotonic ammonium chloride.

In each case, the trypanosome pellet was immediately resuspended in Trifast reagent and then frozen. Subsequently RNA was extracted according to the manufacturer's instructions. The total RNA concentration was then determined using the Qubit 2.0 fluorometer (Invitrogen).

### PolyA+ selection of mRNA, cDNA synthesis and amplification

A detailed step-by-step protocol for the entire procedure is provided in the Supplementary material (Supplementary File S1).

Total RNA was incubated with oligo (dT)-cellulose (Amersham) then the resin was washed before elution of poly(A)+ RNA with water. The RNA was ethanol precipitated and resuspended in water. RNA was then precipitated at −20°C overnight after addition of 30 µl of 5M sodium acetate, 3 µl Glycogen (10 mg/ml), 900 µl of 95% ethanol. The precipitated RNA was then recovered by centrifugation (top speed in the microfuge for 20 min) and resuspended in 6 µl of water.

For all preparations subject to spliced leader priming, cDNA synthesis was done using the Superscript III kit (Invitrogen) according to the manufacturer's instructions. Primers were T3-promoter (5′GCGCGAAATTAACCCTCACTAAAGGGAGA 3′)-tagged oligo-dT (T3(dT)_24_) and random nanomer (T3N9). The synthesized cDNA was purified using the RNAeasy mini-elute kit (Qiagen).

The second strand cDNA was synthesised using 0.05pM SL-4 (5′GATCTACAGTTTCTGTACTAT3′) primer, Phusion polymerase and buffer (Finnzymes). The ds cDNA was visualized by the incorporation of 25 µCi of alpha (^32^P)-dCTP in the reaction, resolved on an 8% polyacrylamide-urea gel, exposed to a Phosphor-imager screen and analyzed using the Fuji FLA7000. Unlabeled ds cDNA was either sent for sequencing (T samples) or amplified (TH samples).

The ds cDNA was also analysed by *in-vitro* transcription using the T3 polymerase reaction mix (MAXIscript, Ambion Inc.). The reaction was carried out using 12 µL of the purified ds cDNA in a mix consisting of 0.5 mM of ATP, CTP, GTP, 50 µCi alpha (^32^P)-UTP, 1× transcription buffer, T3 enzyme mix in a final volume of 20 µl and incubated at 37°C for 10 min. The reaction mix was then treated with DNase1, 1 µl, and continued incubation at 37°C for a further 15 min. The synthesized RNA was then resolved on an 8% Urea acrylamide gel, exposed to a Phosphor-imager screen and analyzed by the Fuji FLA7000.

Trypanosome cDNA sequences were PCR amplified using a T3-promoter primer (5′GCGCGAAATTAACCCTCACTAAAGGGAGA3′) and a 20mer SL primer (5′ACAGTTTCTGTACTATATTG3′). The cDNA was purified using the QIAquick PCR purification kit (Qiagen).

### High throughput sequencing and bioinformatics

The cDNA samples were first fragmented with the Covaris S2 system (Covaris), using the AFA microTube at an Intensity 5, 10% Duty Cycles with 200 cycles per burst for 90 seconds. A quality check on 1 µl of the fragmented sample was done on the BioAnalyzer 2100 (Agilent Technologies) using the High Sensitivity chip. The library was then prepared using the NEBNext Ultra DNA Library Prep Kit for Illumina (New England BioLabs Inc.). Libraries were run on either the Illumina GAII or HiSeq 2000 system for single-end 76 bp or 50 bp reads respectively. Library preparation from poly(A)+ RNA was carried out using the NEBNext Ultra RNA Library Prep Kit for Illumina (New England BioLabs Inc.); and similarly run on the HiSeq 2000 system. All the samples were multiplexed.

The sequence reads were aligned to the reference *T. brucei brucei* TREU 927 genome with Bowtie using the following parameters: –a –best -–strata –v2 –m14 [14]. We extracted all reads that mapped to the annotated mature RNAs (for the amplification experiment) or to coding regions (for the purification experiments). Annotated mature mRNAs in the database do not always include both untranslated regions. For those that did not align, we extracted reads containing sequences of the spliced leader (SL tags) or poly(A) and the T3 promoter, trimmed them, and assigned them to an open reading frame based on their positioning within annotated gene coordinates [15]. The minimum sequence length trimmed was 5 nt. The processing and sorting of the aligned reads was carried out using SAMtools [16] and the read alignment to the genome visualized by Artemis [17], [18].

DESeq was used to identify differentially expressed genes, with a cutoff p-adjusted value of 0.05. Functional category enrichment was carried out using Fisher exact test in R. The heat map was generated in R. Reads per million were calculated using the unique gene list of Siegel et al [8], which excludes all but one gene copy of multigene families. Coding sequence lengths were extracted from TritrypDB. The poly(A)+ mRNA dataset was that from [19], in which reads were mapped to coding regions only.

To plot the average read density along genes, we selected around 1000 short genes of 200–1000b in length, and a similar number of long ones >2.5 kb in length, that had a read coverage of at least 1 on average. PERL scripts were written to extract reads aligning to a window of 0.5% of each gene, which were then normalized to the total read count for the gene, and an average was plotted against the relative location along 1 kb of standardized gene length. Routine calculations were done using Microsoft Excel. Before the calculation of correlation coefficients, the data were log_2_ transformed. If this is not done, a few very abundant mRNAs excessively influence the result.

## Results

### Purification of trypanosomes from blood

To test the effects of trypanosome purification from blood, we compared the two available methods - DEAE cellulose column purification [13] and red cell lysis - with simple centrifugation followed by taking the buffy coat (Table 1). The red cell lysis method does not remove lymphocytes, so would not be suitable at low parasitaemias. The DEAE column does remove a large proportion of the lymphocytes [13], but it takes longer than erythrocyte lysis. Both methods give up to ten times higher trypanosome yields than taking the buffy coat, since many trypanosomes are in the erythrocyte layer after blood centrifugation. However, the buffy coat method does not involve harsh treatment of the parasite, and thus we used it as a reference for the effect of purification methods on the transcriptome.

**Table 1:** Approaches used to analyse trypanosome transcriptomes.

Abbreviation	Trypanosomes	Method
BC	Buffy coat *T. rhodesiense* (Tbr729) from rat blood	poly(A)+ RNA sheared and sequenced with standard Illumina protocol
DE	*T. rhodesiense* (Tbr729) from rat blood purified using DEAE cellulose	poly(A)+ RNA sheared and sequenced with standard Illumina protocol
RL	*T. rhodesiense* (Tbr729) from rat blood purified by erythrocyte lysis	poly(A)+ RNA sheared and sequenced with standard Illumina protocol
Control	Pure culture (Lister427)	poly(A)+ RNA sheared and sequenced with standard Illumina protocol (published results)
T	Pure culture (Lister427)	poly(A)+ RNA; cDNA made by random priming, second strand made using a spliced leader primer.
TH	RNA from pure cultured trypanosomes (Lister427) mixed with a 1000-fold excess of HeLa cell RNA	poly(A)+ RNA; cDNA made using a primer containing a T3 promoter and a random 3′ sequence. Second strand made using a spliced leader primer. Amplification using T3 primer and nested spliced leader primer.

**Figure 1:**
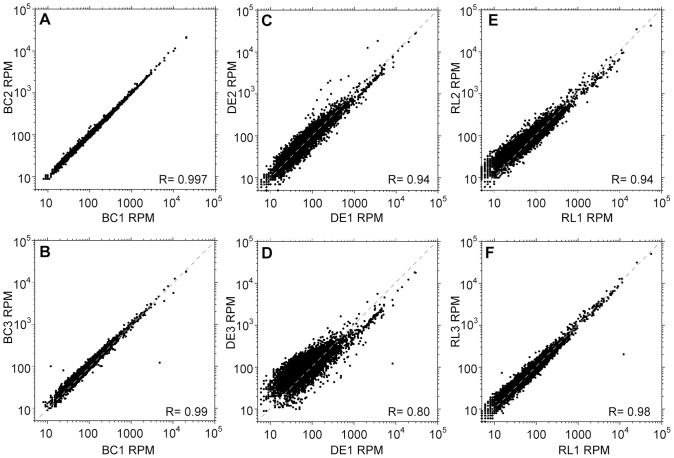
Effect of column purification and erythrocyte lysis on transcriptome profile reproducibility. A–B Buffy coat samples (BC). For each unique open reading frame, the reads per million (RPM) in replicate samples are plotted against each other - replicates 1 and 2 in A, and replicates 1 and 3 in B. The grey dotted line is the regression line for perfect correlation. C–D DEAE-purified samples (DE), plotted as in (A). E–F. Erythrocyte lysis samples (RL), plotted as in (A).

We infected 9 rats with *Trypanosoma rhodesiense* and harvested cells at peak parasitaemia. In all cases, to try to mimic optimal field conditions, an individual animal was bled and the cells prepared as rapidly as possible. For three rats, we made RNA from the buffy coat. For a further three, the trypanosomes were purified using DEAE cellulose. For the remaining three, the erythrocytes were lysed. In each case, the cell pellet was resuspended in Trifast reagent. The triplicate samples were then poly(A) selected, randomly sheared and subjected to RNASeq (Table 1 and Supplementary Table S1). Figure 1 shows the reproducibility of the results for each method. For each open reading frame, we calculated the number of mapping reads. We then extracted the data for individual unique open reading frames (ORFs) in order to eliminate multi-copy ORFs, and calculated the numbers of reads in each unique ORF per million total reads (reads per million, RPM). Correlation coefficients were calculated on log-transformed data, since the count distributions are heavily biased towards lower values. Results from the buffy coat samples (BC) showed correlation coefficients of >0.98 for all three pairwise comparisons (Figure 1A, B), whereas both DEAE purification (DE, Figure 1C,D) and red cell lysis (RL, Figure 1E,F) resulted in more variation (coefficients of 0.80–0.94, and 0.94–0.98 respectively). We have not compared the buffy coat results with those for cultured trypanosomes because we do not have results for cultures of this trypanosome strain. Relative standard deviations (the standard deviation divided by the mean) for individual genes did not correlate with either the mRNA abundance or the half-life (Supplementary Figure S1, A–F) suggesting that neither of these parameters was directly linked to the variation seen.

**Figure 2:**
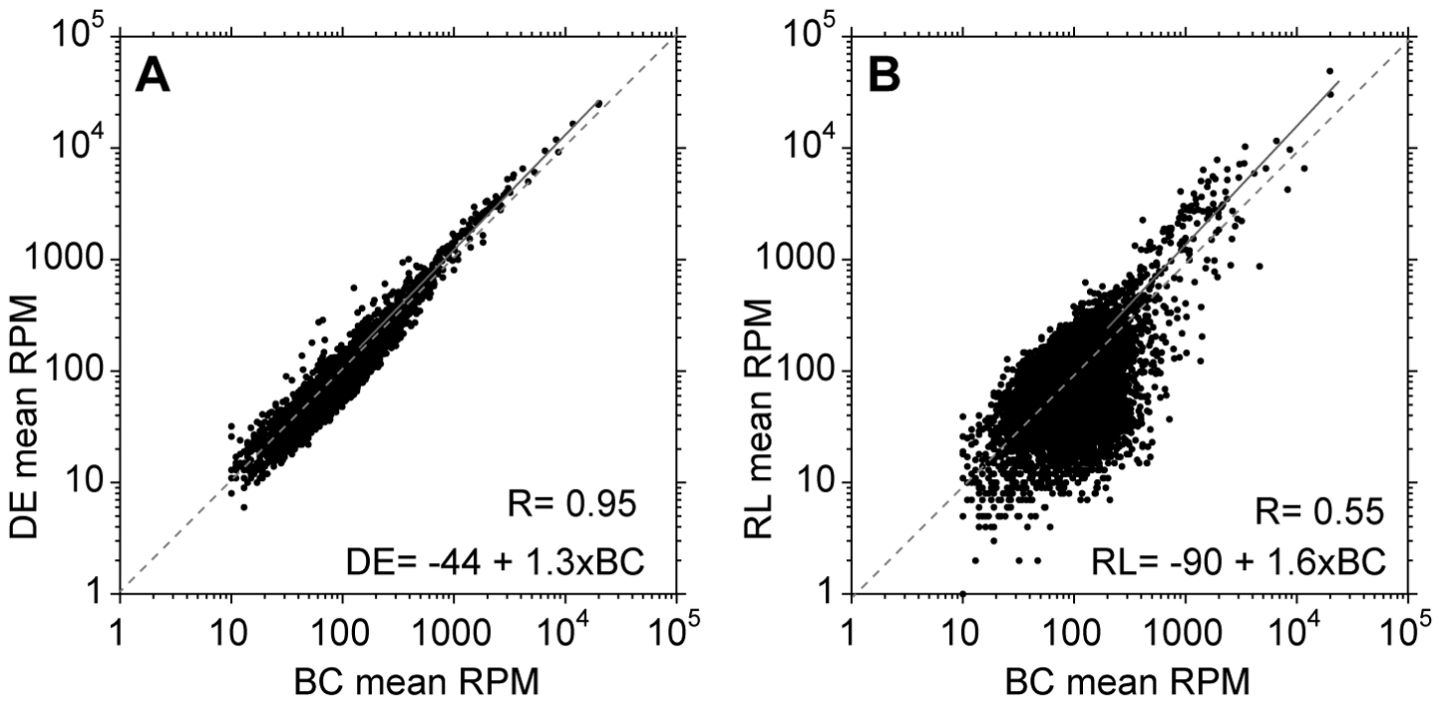
Column purification and erythrocyte lysis cause transcriptome changes. For each condition: buffy coat (BC), DEAE purification (DE) and erythrocyte lysis (RL), and for each unique open reading frame, the arithmetic mean for three independent experiments was calculated. A. Mean counts for DEAE samples compared with those for buffy coat samples. The diagonal dashed grey line is perfect correlation, and the solid grey line shows the linear regression line for which the formula is shown in the bottom right. B. Mean counts for erythrocyte lysis samples compared with those for buffy coat samples. Analysis as in (A).

Next, we compared the transcriptome profiles across the methods (Figure 2). For the DEAE-purified cells results correlated well (Figure 2A); no differentially expressed genes were detected between the two methods, partly because they seem to be quite similar, but also because of the high variability between the DEAE replicates. (For criteria used to detect significant differences, see Methods section.) From the slope of the linear regression line (1.3), DEAE purification tended to decrease the abundances of mRNAs that already were present in low copy numbers in the buffy coat parasites.

Purification by red cell lysis (Figure 2B) caused significant changes in the expression of 60% of genes (see Methods for details). Some functional categories were particularly affected: for example, increases in mRNAs encoding ribosomal proteins, chaperones and mitochondrial metabolic proteins, and decreases in mRNAs encoding protein kinases and cytoskeletal proteins (Supplementary Figure S2). We also looked to see whether mRNAs from some genes were “lost” during purification, considering only genes giving at least 10 reads per million from buffy coat trypanosomes. In the DEAE samples, there were around 30 that fell below 10 reads per million, while after red cell lysis the number lost varied from 43 to 204.

We conclude that if purification is necessary, DEAE cellulose is the method of choice, but some variability will be introduced.

### Selective trypanosome cDNA synthesis and amplification

**Figure 3:**
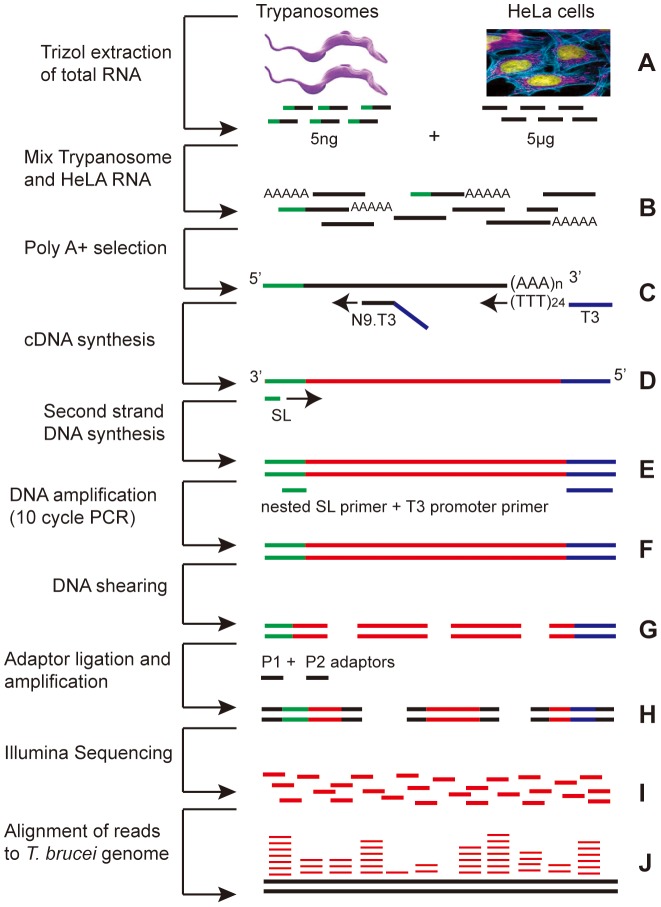
Amplification procedure. For explanation see text. A. RNA extraction; B. Mixing of trypanosome and HeLa RNA, if applicable; C. Location of cDNA primers; D. Location of SL reverse primer; E. Primers used for PCR; F. PCR product; G. Sheared PCR products; H. Sheared DNAs with Illumina adaptors; I. Sequence output; J. Sequences aligned to genome.

For many clinical samples, the numbers of trypanosomes are likely to be too low to allow transcriptome analysis without prior amplification [20], [21], [22], [23], [24](Supplementary Table S2). Even if numbers are adequate, purification may not always be possible under field conditions. We therefore tested methods to amplify parasite mRNA from mixtures of trypanosome and human RNA. To mimic a sample containing 2.5×10^6^ lymphocytes and 10^4^ parasites, we made mixtures containing 5 ng of total *T. brucei* RNA and 5 µg of HeLa cell RNA (Supplementary Table S2; Figure 3A, B).

We first tested methods for cDNA synthesis. The first strand was made using reverse transcriptase primed with a T3 promoter primer bearing, at the 3′ end, (T)_24_ or nine random nucleotides (Figure 3C). To make a *T. brucei*-specific second strand, we tested primers representing various segments of the spliced leader (Figure 3D, Supplementary Figure S3A). Synthesis was measured by including (^32^P)-dCTP in the reaction, and specificity was tested with a negative control of HeLa RNA alone as template. Initial results showed that it was possible to see specific priming only with purified poly(A)+ trypanosome RNA (not shown). Not unexpectedly, selectivity for trypanosome mRNA was strongly affected by the primer sequence and the annealing temperature. Examples of the results for three primers are shown in Supplementary Figure S3B. When we used a primer that included the entire SL (panel SL), the smear from HeLa RNA alone (lane 3) was the same as that obtained when trypanosome RNA was included (lanes 1 and 2), implying insufficient selectivity for trypanosome mRNA. Using shorter, 20mer SL primers (panels SL20 and SL-2), selectivity was seen: the smears for the samples with trypanosome RNA (lanes 5, 6, 9 and 10) gave stronger signals than those for HeLa RNA alone (lanes 7 and 11). The primer concentration was also critical (Supplementary Figure S3C): for example, using 0.5 pM of primer SL-4, there was no difference with or without trypanosome RNA (lanes 1 & 2) whereas with 0.05 pM primer SL-4, five times more product was obtained if trypanosome RNA was present (lanes 3 & 4).

Next, we tested amplification methods and conditions. We attempted linear amplification using T3 polymerase (for explanation, see Introduction) but yields were poor, so we decided to concentrate on PCR. We tested a variety of combinations of spliced leader primers with a T3 promoter primer, with different annealing temperatures and numbers of PCR cycles (Figure 3E). The most specific result was obtained using a “nested” PCR strategy. After cDNA synthesis, the second strand was primed with the 20mer primer SL-4, which terminates 4 nt upstream of the 3′-end of the spliced leader. With authentic trypanosome mRNAs, the first four nt synthesised will be ATTG - the last 4 nt of the spliced leader. This should be absent in most mis-primed HeLa cDNAs. Amplification was then done with the T3 primer and a 20mer primer (“SL20”) that terminated at the end of the spliced leader. This primer combination will only work if the first four nt after the SL-4 primer really were ATTG, providing an additional level of specificity. PCR results using the optimal primer combination and conditions (see Methods) are illustrated in Supplementary Figure S3D: with this combination, we usually obtained at least twice as much PCR product with the trypanosome-HeLa mix as with HeLa RNA alone.

### Effect of spliced leader priming and amplification on transcriptome measurements

We now analysed the effects of spliced leader priming and amplification on the measured transcriptome. The cDNAs obtained by spliced leader priming alone, using poly(A)+ RNA as substrate (T in subsequent figures and tables) or using 1000∶1 HeLa mixtures as described above (TH in the figures and tables) was sheared, Illumina adaptors were added, and the resulting fragments amplified and sequenced according to the standard Illumina protocol (Figure 3F–I). In a first experiment duplicate RNA preparations were sequenced using the Illumina GAII sequencer. Later, two more replicates were analysed using the Illumina HiSeq 2000, with duplicate random-shear controls sequenced in the same way. All reads were aligned to the trypanosome genome. Statistics for these experiments are shown in Table 2 and results are in Supplementary Table S3. In the two replicates in first amplification experiment, TH1 and TH2, only about 2%, , of amplified reads aligned to the trypanosome genome (Table 2), which is roughly 20-fold enrichment. In the second experiment (replicates TH3 and TH4), enrichment was 10 times better, with 20% of reads aligning (Table 2). It is possible that the use of HiSeq technology in the second experiment increased the percentage of mapped reads, but variations in the amplification efficiency could also have contributed. We extracted all reads that mapped to the predicted mature RNAs available in TritrypDB (CDS). In addition, we extracted reads containing the spliced leader (SL tags) or poly(A) and the T3 promoter, and assigned them to an open reading frame based on their positions in the genome. Most analyses were done with all reads added together.

**Table 2:** Statistics for the sequencing runs - T and TH.

Sample	T1	T2	T3	T4	TH1	TH2	TH3	TH4
Total reads	17,790,273	14,796,988	47,539,876	59,769,712	17,909,805	20,350,255	30,889,345	34,076,525
Tb gene	10,348,505	8,379,566	33,441,648	42,639,172	241,924	409,262	4,585,299	6,508,792
SL/pA-T3 tags	67,355	47,367	6,143,230	7,228,812	100,964	177,369	869,722	1,403,415
Total Tb	10,415,860	8,426,933	39,584,878	49,867,984	342,888	586,631	5,455,021	7,912,207
% aligned to Tb	59%	57%	83%	83%	1.9%	2.9%	18%	23%
Tb gene (unique)	5,642,112	5,658,357	22,036,845	28,335,223	151,006	258,159	3,179,912	4,391,140
SL tags (unique)	nd	nd	3,503,330	4,136,515	nd	nd	465,715	744,833
pA/T3 tags (unique)	nd	nd	91,301	117,950	nd	nd	134,490	205,786
no genes <1 RPM	25	97	11	32	1761	1846	17	15
no genes <10 RPM*	489	917	646	1154	2486	2796	2038	2239

Total reads that matched to the *T. brucei brucei* TREU927 genome (Tb) are shown. The top 5 rows include reads that mapped to variant surface glycoproteins and some other multi-gene families. The bottom 4 rows consider only the list of unique genes [8] and the two rows “no. genes” consider only genes that had at least 10 RPM in the randomly sheared samples. For samples 1 and 2, SL tags and poly(A) tags were extracted together.

**Figure 4:**
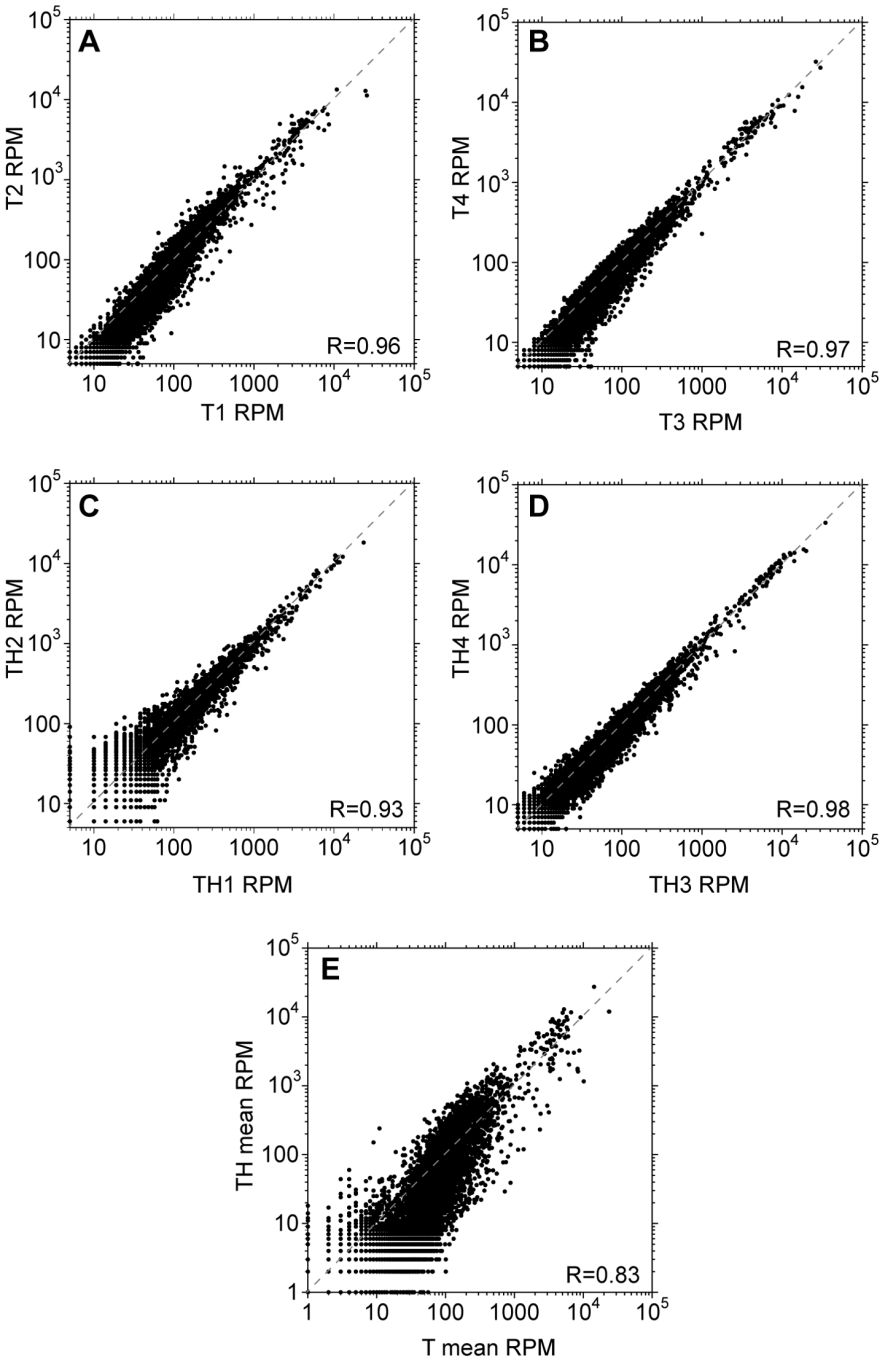
Reproducibility of spliced leader priming and amplification. Transcriptomes are represented as in Figure 1; results for individual experiments are compared. A. Reproducibility after spliced leader priming without amplification (sample T1 versus sample T2). B. Reproducibility after spliced leader priming without amplification (sample T3 versus sample T4). C. Reproducibility after spliced leader priming and amplification, using trypanosome RNA mixed with a 1000-fold excess of HeLa RNA (sample TH1 versus sample TH2). D. Reproducibility after spliced leader priming and amplification (sample TH3 versus sample TH4). E. Amplification affects read counts with a loss of some genes. The mean read counts for all unamplified samples (T1–T4) are plotted against those for all TH samples (TH1–TH4).

Results from technical replicates using spliced leader priming alone, with no amplification, correlated well with each other although as usual, reproducibility was poor for ORFs with low read counts (Figure 4A, B). Spliced leader tagging methods described previously were also reproducible [10]. Although the correlation between the replicates in the first amplification experiment (TH1 and TH2) was reasonable (Figure 4C), the scatter for many genes was unacceptable and over 1700 genes were not represented at all. This is almost certainly due to the very low number of mapped reads (Table 2), and indicates that data should only be used if several million mapped reads are obtained. Results from the second amplification experiment (replicates TH3 and TH4, Figure 4D) correlated well, with less scatter at low RPM values compared to the first experiment because the number of mapped reads was roughly 10-fold higher (Table 2). Results from amplified cDNA correlated to some extent with those from unamplified: Figure 4E shows the results for all four experiments, but the correlation was the same when only samples TH3, TH4, T3 and T4 were considered.

**Figure 5:**
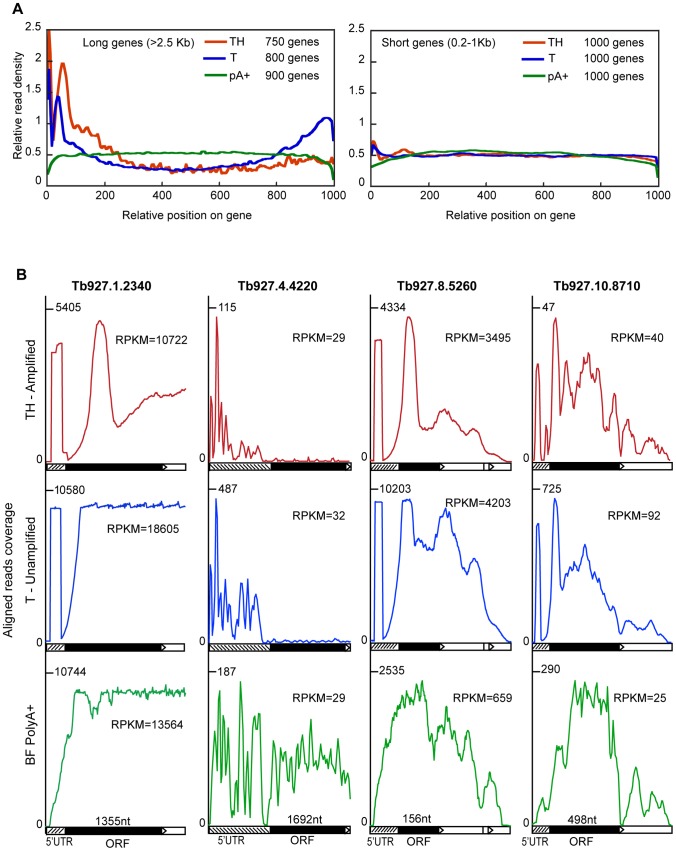
Spliced leader priming leads to unevenly distributed reads. A. Reads were mapped across mRNAs, then each mRNA was normalised to a standard length of 1000. Around 1000 mRNAs of short (less than 1 kb) or long (>2.5 kb) lengths were selected, and the average read densities across the mRNAs were plotted for unamplified poly(A)+ mRNA (pA+), and samples T4 (spliced leader primed, unamplified) and TH4. B. The read distributions over four different genes are shown, using data from samples T4 and TH4. For each, the 5′-end of the mRNA is on the far left, and the open reading frame is filled black. The maximum read density is on the left-hand scale and the total number of reads is also indicated.

Future discussion will focus mainly on the second experiment (samples T3, T4, TH3 and TH4), because the number of genes with sufficient mapped reads to estimate the mRNA abundance was acceptable for the amplified samples. When randomly sheared mRNA is subjected to RNASeq, the reads should be distributed evenly throughout the open reading frame [2]. We expected spliced leader priming to give 5′ bias [11] even though the cDNA was fragmented before library construction. Figure 5A shows the profiles of read densities along a standardized gene length of 1 kb. For short genes (between 200 bp–1 kb) the reads were generally uniformly distributed with a slight peak at the 5′ end for both amplified and unamplified samples, compared to a randomly sheared sample. Genes of 1–3 kb gave similar results (not shown) but the sample size was much larger than from the short and long genes, which could have concealed many variations. In contrast, there was a big bias towards the 5′ ends for long genes (>2.5 kb); in the case of the un-amplified sample, there was another peak at the 3′ end (Figure 5A), even though random primers, as well as oligo d(T), were used for cDNA priming. We also noticed a double peak at the 5′-ends of long genes, which was not so pronounced for shorter ones. The first peak corresponded to reads with the SL primer and was limited in length to ∼30 nt: this is the read length of 50 nt minus the 20mer SL sequence used for priming. Perhaps fragmentation was not so frequent near the ends and most end fragments carried the SL sequence, hence the drop between the two peaks.

Figure 5B shows alignments for four genes with different patterns. For the highly abundant alpha tubulin mRNA Tb927.1.2340 in the spliced leader primed samples (central panel - T, unamplified), we found a strong peak near the 5′ end but otherwise, fairly uniform reads throughout. After amplification (top panel), distortions arose. In contrast, for Tb927.4.4220, a gene of similar ORF length but with much lower mRNA abundance and longer 5′-UTR, the spliced-leader primed reads were restricted to the 5′-UTR. RNA from this gene would hardly be detected if only CDS reads were analysed. For the tiny ORF Tb927.8.5260, a strong 5′ peak was in the spliced leader primed sample but not the random shear control, and a rather similar pattern was seen for the longer gene Tb927.10.8710.

For randomly sheared mRNAs, the read counts increase with open reading frame length. The mRNA abundance is therefore proportional not to the read counts, but to the read density, which is expressed as reads per million reads per kb of open reading frame (RPKM). As expected, when we calculated the RPKM for the spliced leader primed samples we detected a clear bias against longer transcripts (Supplementary Figure S4 C–F) illustrating what we observed above in the average read density profiles for long genes.

**Figure 6:**
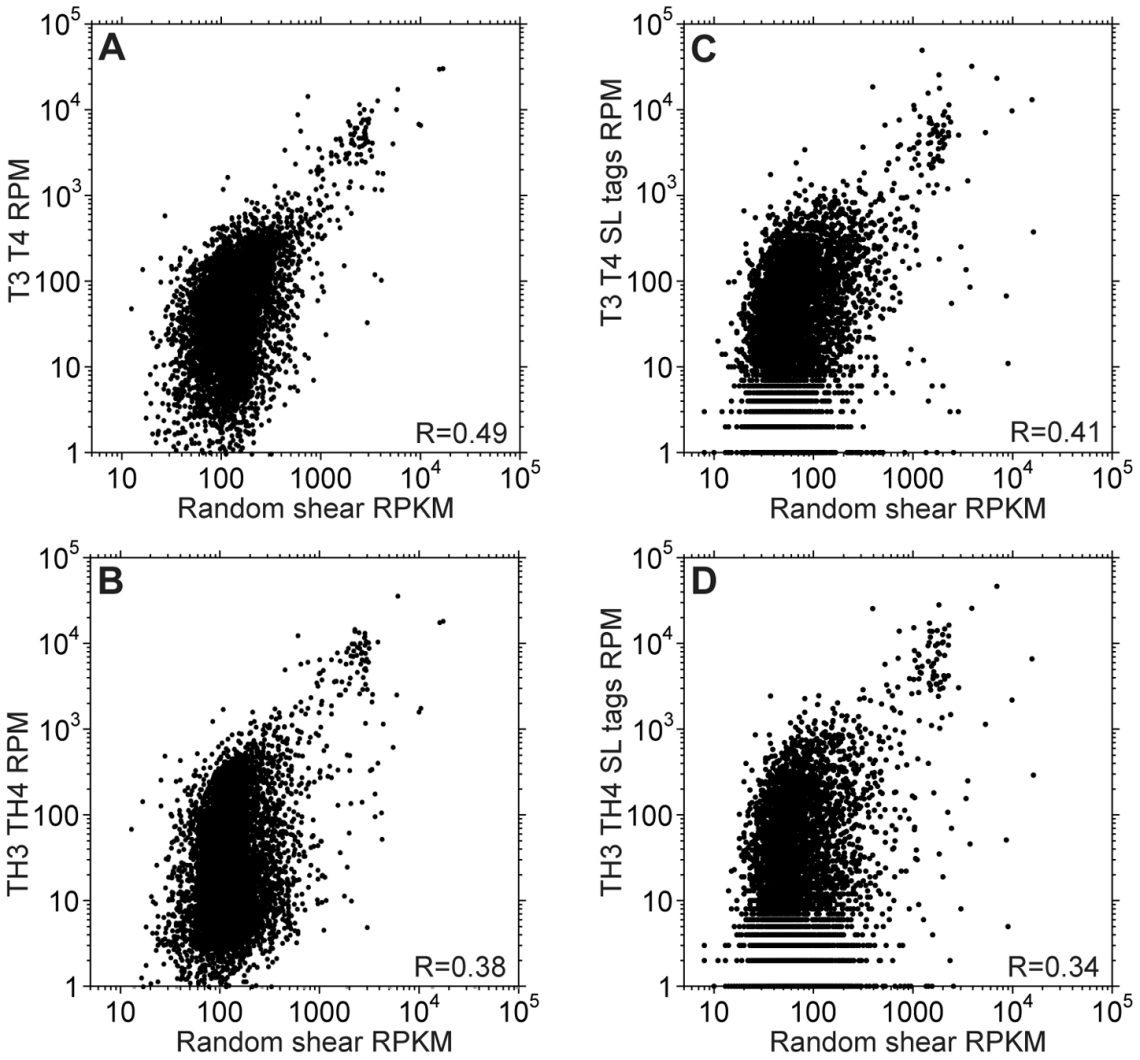
Transcriptomes obtained by spliced leader priming differ from those obtained using randomly sheared RNA. The results from spliced leader-primer cDNA synthesis (T) or from amplified samples (TH) are compared with those from control randomly sheared poly(A)+ RNA. Each result is the mean of two independent measurements (T3 and T4, and TH3 and TH4 for the spliced leader primed transcriptomes). Only unique CDSs with and average of at least 10 RPM in the control are shown. Correlation coefficients were calculated after log_2_ transformation of the data. A. Average RPM for spliced leader primed samples (all reads) vs. control RPKM (reads per million per kilobase of CDS). Without log transfomation, R^2^ is 0.84 for the unamplified total reads; however, exclusion of the 100 most abundant mRNAs (out of 6652) reduces the correlation to 0.39. B. Average RPM for spliced leader primed, amplified samples (all reads) vs. control RPKM. C. Average RPM for spliced leader primed samples (spliced leader-containing reads only) vs. control read density, RPKM. D. Average RPM for spliced leader primed, amplified samples (spliced leader-containing reads only) vs. control read density, RPKM.

We now quantitatively compared the results from spliced leader primed RNAs with those from classical RNA-Seq of randomly-sheared RNA. The latter is currently the standard method for mRNA quantitation, although it too is likely to have some biases. Since we already knew that our spliced-leader-primed reads were heavily 5′ biased, we first corrected the classical RNASeq data for mRNA length (see Figure 6 legend). In all graphs most mRNAs formed a dense cloud with little apparent correlation, while a small minority was abundant in both datasets (Figure 6). Results for the total counts (Figure 6A, B) correlated rather better than those for SL tags (Figure 6C,D). Correlations for each gene excluding SL and poly(A)/T3 tags were similar (not shown), which is not surprising because there were about six times more gene reads than SL tags (Table 2). Since our amplified and unamplified spliced leader-primed results correlated better with each other (Figure 4F) than with the classical RNASeq, the major cause of the difference between the T and TH samples and randomly sheared pure RNA must have been the spliced leader priming. We found no GC content bias from amplification.

Nilsson et al. have previously used spliced-leader priming followed by RNA Seq to measure mRNAs in purified trypanosomes [10]. Their method differed somewhat from ours. For example, they used a second-strand primer that covered the entire spliced leader; we could not do this because the long primer gave too much background priming on HeLa cDNA (Supplementary Figure S3B). Nevertheless, when they compared their SL tag counts with those obtained from total (not SL-primed) cDNA, the dot plots looked very similar to ours (compare Figure 6E with Supplementary Figure S9 in [10]). This suggests that in their experiments also, SL priming introduced distortions.

We conclude that synthesis of the second cDNA strand with a spliced leader primer, followed by amplification, is sufficiently reproducible to allow comparison of different samples so long as they are all treated in the same way. However, SL priming gives results that differ substantially from those obtained using the standard protocol with randomly sheared mRNA.

## Discussion

To analyse the transcriptomes of field samples, the choice of the most appropriate method will depend on the number of parasites present relative to host cells, and the total number of parasites in the sample. A decision can be made on a sample-to-sample basis if parasitaemias and lymphocyte counts can be checked prior to sampling.

We first assume an experimental scenario with a parasitaemia of 4×10^5^/ml and a lymphocyte count of 4×10^6^/ml. This could arise in *T. b. rhodesiense* patient samples. An average lymphocyte yields 5 times more mRNA than a trypanosome (see Supplementary Table S3). Use of whole blood gives maximum trypanosome yield with minimal handling. If RNA were prepared from whole blood, and sequenced on a single lane, approximately 1 million reads might map unambiguously to trypanosome coding regions (Supplementary Table S2) (This assumes that globin mRNA is removed prior to library preparation.) 1 million reads is just about the lowest threshold for transcriptome analysis: most coding regions would map around 50 reads [23]. This would also yield the total lymphocyte transcriptome as a by-product, although this will give limited information since it will come from a complex mixture of cell types. For samples with parasite∶lymphocyte ratios of at least 1∶10, the variability that ensues from low read counts has to be weighed against the variability of results, and the decrease in low-abundance mRNAs detected, that are introduced by column purification. An expensive alternative would be to apply the sequencing library to several lanes in order to get more reads.

If parasite∶lymphocyte ratios are lower than 1∶10, either the parasites must be purified, or the parasite mRNA must be specifically amplified. Standard Illumina library preparations work best with at least 50 ng of mRNA sample, which can be obtained from about 2×10^6^ trypanosomes (Supplementary Table S2). If that number is available, column purification is a good option although the results will be less reproducible than for unpurified samples. We already saw variations under ideal lab conditions, but in a field lab purification times might be longer and the temperature could be higher, both of which could have deleterious effects. Samples with fewer parasites could be pooled prior to RNA preparation, which might to some extent compensate for the variability introduced by column purification.

For samples containing less than about 5×10^5^ parasites, amplification of some sort will be unavoidable. We were able to achieve up to 200-fold enrichment of trypanosome sequences from a 1000∶1 HeLa∶trypanosome mixture. This gave ample coverage of the transcriptome starting with only 5 ng of trypanosome RNA. The reads obtained were not directly proportional to mRNA abundance, but were instead strongly influenced by the spliced leader priming and also, to a lesser extent, by the PCR amplification. Despite this, the method was reproducible, especially when 200-fold enrichment was achieved. This indicates that the distortions that were seen must have been due to sequence-specific effects such as blockage of reverse transcriptase by secondary structures, PCR bias towards shorter amplicons, and long 5′-untranslated regions. Because the results from the method are reproducible, data from different samples can be compared. Ideally, all samples should be treated in the same way, with at least 3 replicates per condition; under those conditions relative mRNA levels for each gene could be calculated and differentially expressed genes detected. Alternatively, the results could be converted into approximate “real” mRNA abundances using correction factors. These would need to be measured in individual labs, since they will be affected by the sequencing platform, the precise amplification and library building conditions, and the database version used for the alignments.

Spliced leader priming of mixed human/trypanosome poly(A)+ mRNA has the advantage of starting with quite large RNA amounts, which are easy to handle. The alternative, for samples containing at least 10^4^ parasites, would be to purify the parasites using a column, then make the library using a method or kit designed for picogram amounts of mRNA. There have been relatively few independent evaluations of these amplification protocols for transcriptome quantitation, but all of them cause method-specific distortions. The consensus so far is that if amplification is necessary, the results for different samples can only be compared if the same method is used throughout ([3], [4], [5] and http://openwetware.org/wiki/BioMicroCenter:RNAseq).

Our method for spliced leader priming and amplification of trypanosome mRNA should be suitable for any sample containing at least 10^4^ trypanosomes, and could be particularly useful if conditions do not allow immediate and rapid column chromatography. In principle, samples from *T. b. gambiense* sleeping sickness patients could be analysed. The same principle could be applied for any other organism in which the mRNAs carry a spliced leader: our results show, though, that careful optimisation of the spliced leader primers, ratios and annealing temperature will be necessary. Once this is achieved, the method might be used to analyse gene expression in samples from *Trypanosoma congolense* or *Trypanosoma vivax* infected cattle, for intracellular kinetoplastids, or samples from infected vectors.

## Supporting Information

Figure S1 The variability in results from purified trypanosomes does not correlate with mRNA abundance or half-life. A–F. For each unique open reading frame, and for all three methods, the relative standard deviation of RPM was calculated by dividing the standard deviation by the mean The relative standard deviations (relative SD) were then plotted against either RPM (A–C) or against the mRNA half-life in cultured bloodstream forms (A. Fadda, ZMBH, manuscript in preparation) (D–F). A, D: buffy coat (BC): B, E: DEAE (DE); C, F: erythrocyte lysis (RL). All half-lives over 240 min were arbitrarily set to 240 min.
(TIF)

Figure S2 The erythrocyte lysis procedure preferentially affects specific functional categories of genes. All unique open reading frames were manually placed in functional categories, and significantly regulated genes were found. The enrichment of specific categories in the mRNAs that were higher or lower in buffy coat (BC) or DEAE-purified (DE) parasites, relative to erythrocyte lysis, is displayed as a heat map. “pvalue” refers to the p-values from Fisher exact test.
(PDF)

Figure S3 Conditions for second strand synthesis and PCR amplification of cDNA. A. The splice leader sequence primers, SL, full length splice leader, SL20, primer spanning the first 20 bp (from the 3′end) of the full-length, SL-2, primer spanning 20 bp after the first 2 bases (TG) of the full-length and SL-4, primer spanning 21 bp after the first 4 bases (ATTG). B. A second strand synthesis reaction was done using various splice leader primers and Phusion polymerase, incorporating α^32^P-dCTP, at 95°C for 2 min, 50°C for 3 min and 72°C for 5 min. The ds cDNA was run on 8% Urea-polyacrylamide gel and visualised by phosphorimaging. The SL primer gave no preferential synthesis of trypanosome cDNA (compare lane 3 with lanes 1 and 2). The SL20 primer was somewhat better (compare lane 7 with lanes 5 and 6) and the SL-4 primer was best (compare lane 11 with lanes 9 and 10). C. The amount of primer used is important for selectivity. Second strand synthesis was done as in (B) but with two different primer concentrations. D. Nested PCR using SL20 or SL-2. The cDNA was made without labelling, then amplified with 10 cycles of 95°C for 1 min, 60°C for 3 min and 72°C for 5 min. This time the double-stranded cDNA was not labelled, but radioactive dCTP was included in the PCR reaction. Under these conditions the SL20 primer reproducibly yielded a TH∶H ratio of 2–3 to 1.
(TIF)

Figure S4 The effects of mRNA length and abundance on spliced leader priming and amplification. A, B. The variability in the reads is not lower for abundant mRNAs. For preparations without (A) and with (B) amplification, the standard deviation for the RPM four experiments was divided by the mean RPM to get a relative standard deviation. This was then plotted (y axis) against the mean RPM for each gene. C–F. Spliced leader priming results are biased against longer mRNAs. For each open reading frame, the mean RPKM is plotted against the length of ORF+5′-UTR. C. TH (amplified) samples; D. T (unamplified) samples. E. Standard method: the RNA was randomly sheared before library preparation. No length bias is seen. F. Overlay: The TH (red) and T (blue) samples look quite similar and clearly differ from the randomly sheared sample (Green).
(JPG)

File S1 Detailed protocol for trypanosome mRNA spliced leader priming and amplification.
(DOCX)

Table S1 Sequencing results for different trypanosome purification methods. The first sheet shows reads per million reads for unique genes. The sums of reads for all unique genes were used for normalisation. BC: buffy coat; DE: DEAE-purified trypanosomes; RL: trypanosomes obtained by red cell lysis. Sheet 2 shows results after removal of all genes which, on average, gave fewer than 10 rpm in the buffy coat samples. Means, standard deviations (SD) and relative standard deviations (SD/mean) are also shown.
(XLSX)

Table S2 Calculations of expected RNA amounts in lymphocytes and trypanosomes, and amounts needed for sequencing: details and sources of the information.
(DOCX)

Table S3 Sequencing results for spliced leader priming and amplification. Sheet 1 shows the raw reads for all genes. Gene: reads in the annotated mature RNA. SL: reads that contained SL sequence. pA: reads that contain poly(A). Sum: sum of all reads for a particular gene in that particular replicate. T - spliced leader primed mRNA, no amplification; TH: 1∶1000 trypanosome to HeLa mixture, spliced leader primed and amplified. 1,2 3 and 4 refer to the replicates. Sheet 2: reads per million reads (RPM) for unique genes. Sheet 3: Factors (CF) for converting amplified RPM to the equivalent RPM from randomly sheared poly(A)+ mRNA. More details are in the text inset. Sheet 4: Comparison of SL tag reads with the equivalent RPKM from randomly sheared poly(A)+ mRNA.
(XLS)
