# Supplementary figures and images for: Methods to Determine the Transcriptomes of Trypanosomes in Mixtures with Mammalian Cells: The Effects of Parasite Purification and Selective cDNA Amplification
Source: PLoS Negl Trop Dis. 2014 Apr 17;8(4):e2806. doi: 10.1371/journal.pntd.0002806 (PMC3990519; doi:10.1371/journal.pntd.0002806)

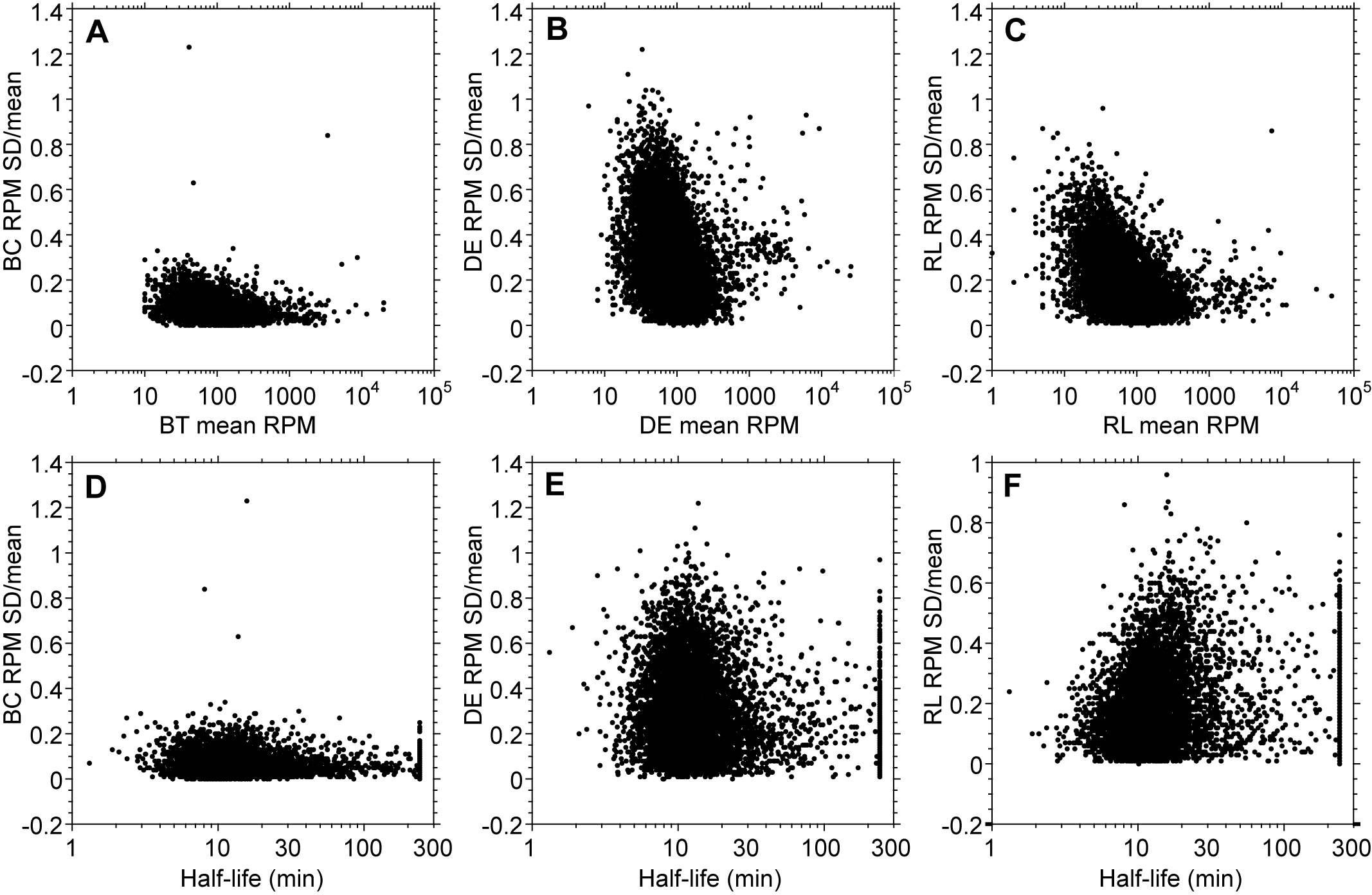

Supplement: Figure S1 — The variability in results from purified trypanosomes does not correlate with mRNA abundance or half-life. A–F. For each unique open reading frame, and for all three methods, the relative standard deviation of RPM was calculated by dividing the standard deviation by the mean The relative standard deviations (relative SD) were then plotted against either RPM (A–C) or against the mRNA half-life in cultured bloodstream forms (A. Fadda, ZMBH, manuscript in preparation) (D–F). A, D: buffy coat (BC): B, E: DEAE (DE); C, F: erythrocyte lysis (RL). All half-lives over 240 min were arbitrarily set to 240 min. (TIF) [file pntd.0002806.s001.tif]

Color Key

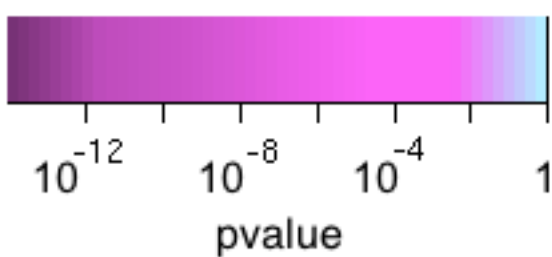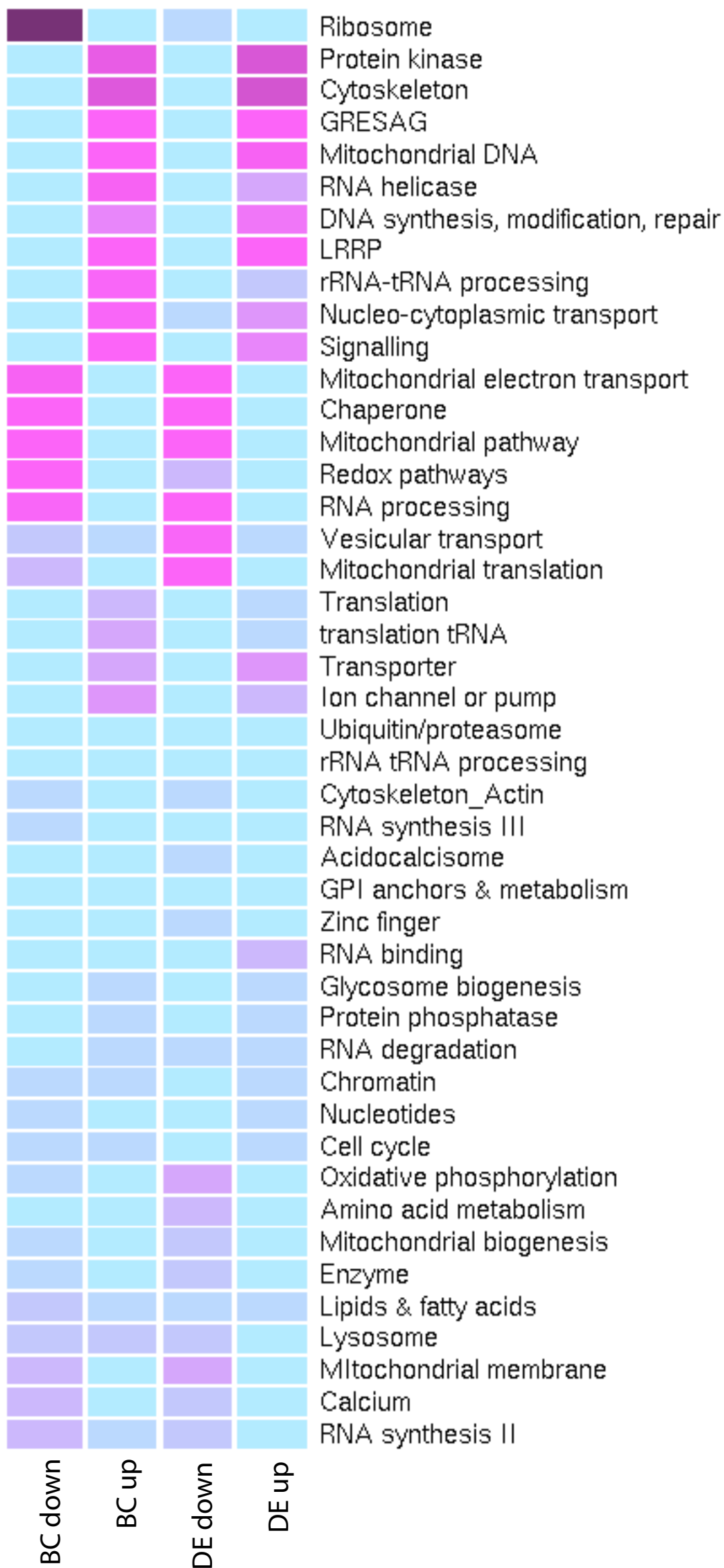

Supplement: Figure S2 — The erythrocyte lysis procedure preferentially affects specific functional categories of genes. All unique open reading frames were manually placed in functional categories, and significantly regulated genes were found. The enrichment of specific categories in the mRNAs that were higher or lower in buffy coat (BC) or DEAE-purified (DE) parasites, relative to erythrocyte lysis, is displayed as a heat map. “pvalue” refers to the p-values from Fisher exact test. (PDF) [file pntd.0002806.s002.pdf]

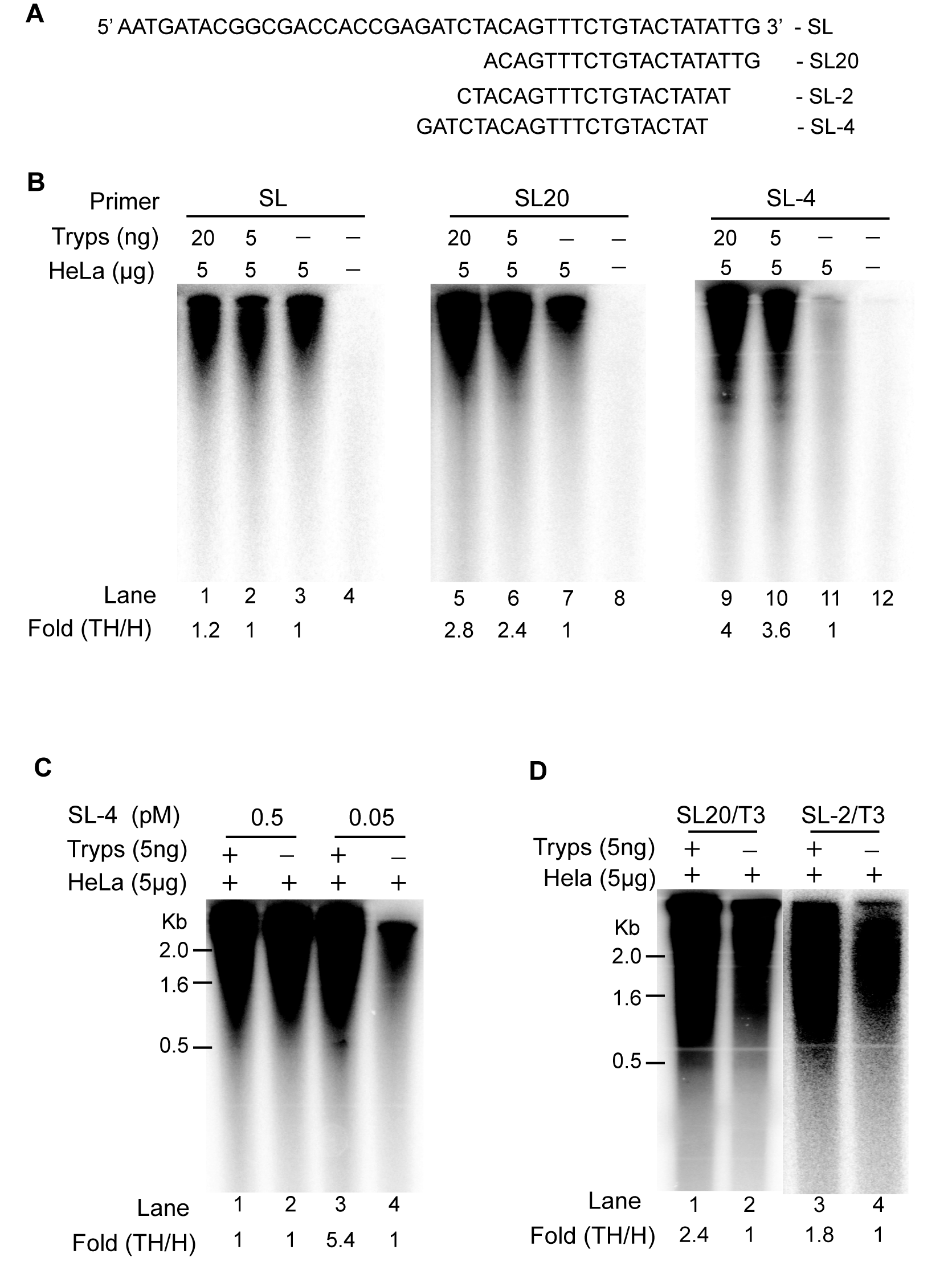

Supplement: Figure S3 — Conditions for second strand synthesis and PCR amplification of cDNA. A. The splice leader sequence primers, SL, full length splice leader, SL20, primer spanning the first 20 bp (from the 3′end) of the full-length, SL-2, primer spanning 20 bp after the first 2 bases (TG) of the full-length and SL-4, primer spanning 21 bp after the first 4 bases (ATTG). B. A second strand synthesis reaction was done using various splice leader primers and Phusion polymerase, incorporating α32P-dCTP, at 95°C for 2 min, 50°C for 3 min and 72°C for 5 min. The ds cDNA was run on 8% Urea-polyacrylamide gel and visualised by phosphorimaging. The SL primer gave no preferential synthesis of trypanosome cDNA (compare lane 3 with lanes 1 and 2). The SL20 primer was somewhat better (compare lane 7 with lanes 5 and 6) and the SL-4 primer was best (compare lane 11 with lanes 9 and 10). C. The amount of primer used is important for selectivity. Second strand synthesis was done as in (B) but with two different primer concentrations. D. Nested PCR using SL20 or SL-2. The cDNA was made without labelling, then amplified with 10 cycles of 95°C for 1 min, 60°C for 3 min and 72°C for 5 min. This time the double-stranded cDNA was not labelled, but radioactive dCTP was included in the PCR reaction. Under these conditions the SL20 primer reproducibly yielded a TH∶H ratio of 2–3 to 1. (TIF) [file pntd.0002806.s003.tif]

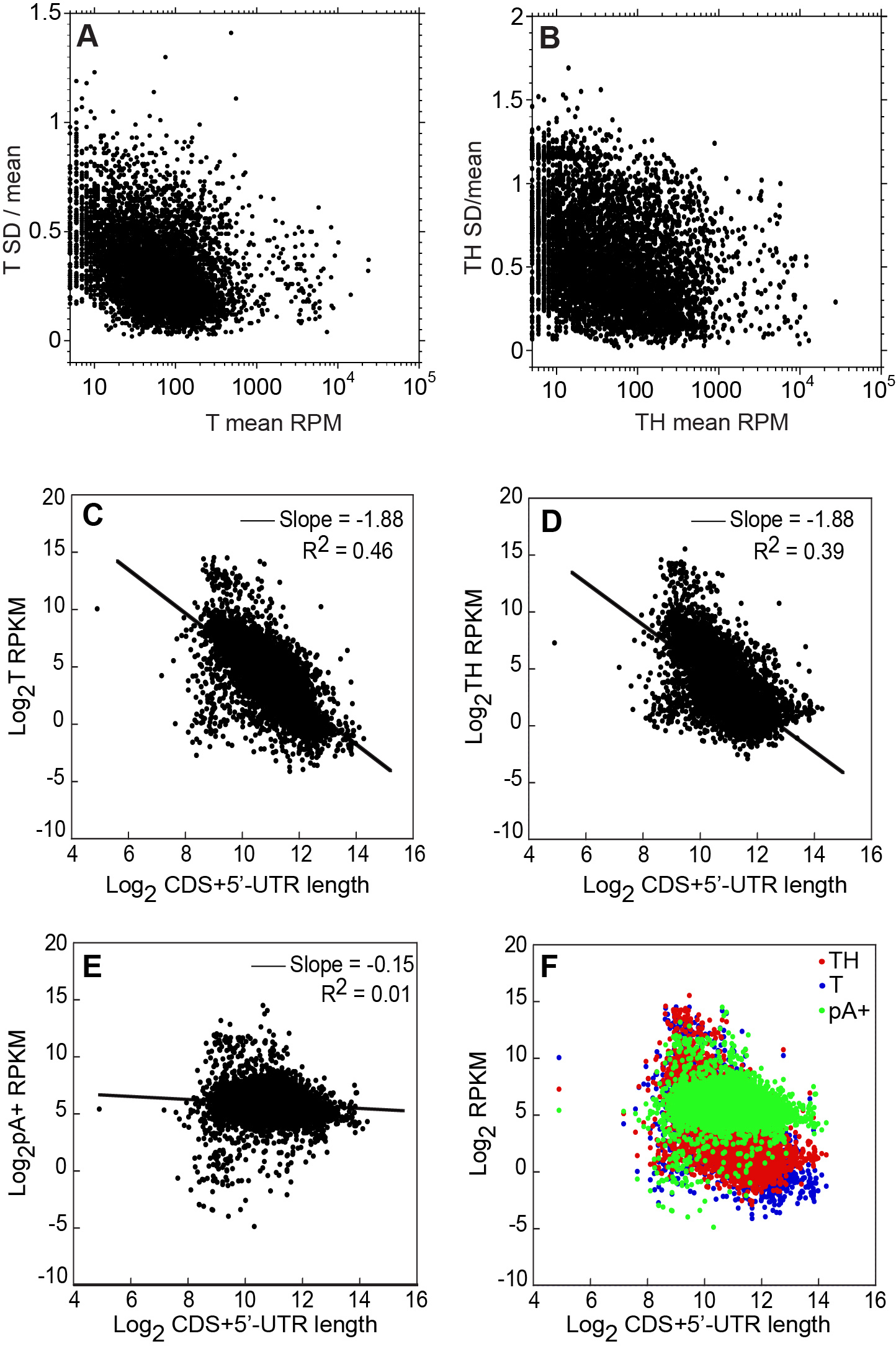

Supplement: Figure S4 — The effects of mRNA length and abundance on spliced leader priming and amplification. A, B. The variability in the reads is not lower for abundant mRNAs. For preparations without (A) and with (B) amplification, the standard deviation for the RPM four experiments was divided by the mean RPM to get a relative standard deviation. This was then plotted (y axis) against the mean RPM for each gene. C–F. Spliced leader priming results are biased against longer mRNAs. For each open reading frame, the mean RPKM is plotted against the length of ORF+5′-UTR. C. TH (amplified) samples; D. T (unamplified) samples. E. Standard method: the RNA was randomly sheared before library preparation. No length bias is seen. F. Overlay: The TH (red) and T (blue) samples look quite similar and clearly differ from the randomly sheared sample (Green). (JPG) [file pntd.0002806.s004.jpg]
