# Supplementary material for: Methods to Determine the Transcriptomes of Trypanosomes in Mixtures with Mammalian Cells: The Effects of Parasite Purification and Selective cDNA Amplification
Source: PLoS Negl Trop Dis. 2014 Apr 17;8(4):e2806. doi: 10.1371/journal.pntd.0002806 (PMC3990519; doi:10.1371/journal.pntd.0002806)
Supplement: File S1 — Detailed protocol for trypanosome mRNA spliced leader priming and amplification. (DOCX) [file pntd.0002806.s005.docx]

**Amplification of Trypanosome cDNA**

**A. Total RNA extraction**

1. Resuspend cellular pellet in 1ml of Trifast reagent and either use immediately or freeze at -20°C for later use.
2. Add 200µl of chloroform to the Trifast cell suspension, vortex vigorously and incubate at room temperature for 3min.
3. Centrifuge the mix at 10000xg for 10min at 4°C and transfer aqueous phase (approximately 500µl) into a 1.5ml eppendoff lobind tube.
4. Add 500µl of isopropanol, mix thoroughly by inverting the tube then incubate at room temperature for 10min to allow for RNA precipitation.
5. Centrifuge at 10000xg, 4°C for 20min and then wash the RNA pellet with 1ml of 75% ethanol.
6. Centrifuge again at 10000xg for 5min, remove the residual ethanol and air-dry the RNA pellet.
7. Dissolve the RNA pellet in 20µl of nuclease free water. Draw 1µl for quantifying the total RNA concentration using the Qubit2.0 fluorometer (Invitrogen).

**B. PolyA+ selection of mRNA**

**Pretreatment of oligo-dT cellulose**

1. Add 5ml of 0.1M NaOH to 100mg oligo-dT cellulose in 15ml tube. Invert on a rotatory mixer for 10min at room temperature. Centrifuge at 3000xg for 5min.
2. Wash cellulose pellet with 15ml RNA binding buffer (20mM HEPES, pH7.4, 5mM EDTA, pH 7.4, 0.4% SDS, 500mM NaCl) centrifuging each time at 3000g for 5min.
3. Resuspend the oligo-dT in 1ml of RNA binding buffer and store the pretreated at 4°C

**Procedure**

1. Resuspend the total RNA in 500µl of RNA denaturing buffer (20mM HEPES, pH7.4, 10mM EDTA, pH7.4, 1% SDS) and incubate it at 65°C for 10min then on ice for 2min.
2. Add 750µl of RNA dilution buffer (20mM HEPES, pH7.4, 800mM NaCl) and 100µl of the pretreated oligo-dT cellulose; and then place the tube on a rotatory mixer for 15min at room temperature.
3. Centrifuge the mix at 10000xg for 2min, remove the supernatant and wash the cellulose pellet 2 times with RNA washing buffer (20mM HEPES, pH7.4, 5mM EDTA, pH7.4, 0.5% SDS), centrifuging each time at 10000xg for 2min.
4. Incubate the cellulose in 150µl of RNAse free water, invert at room temperature for 5min, then centrifuge at 10000xg for 2min.
5. Transfer the supernatant to a 1.5ml eppendorff lobind tube and repeat the incubation in RNAse free water once, then pool the samples to 300µl total volume.
6. Precipitate the RNA by adding 30µl of 5M NaAc, 3µl Glycogen (10mg/ml), 900µl of absolute ethanol, placing at -20°C overnight.
7. The precipitated RNA is then recovered by centrifugation at 10000g for 20min and resuspending the RNA in 6µl of RNAse free water.

**C. First strand cDNA synthesis**

1. RNA denaturation: Set up the reaction mix tabulated below;

| Reagent | Conc. | Vol. |
| --- | --- | --- |
| RNA Mix |  | 6µl |
| T3(dT)_24_ | 5pM | 1µl |
| T3N9 | 5pM | 1µl |
| RNAse Out (Inviitrogen) | 40U | 1µl |

*T3-promoter (5’GCGCGAAATTAACCCTCACT AAAGGGAGA 3’)*

1. Incubate the mix at 65°C for 10min in a thermocycler, then snap cool on ice for 4min.
2. Add 11µl of the cDNA synthesis mix tabulated below;

| RT buffer | 10x | 2µl |
| --- | --- | --- |
| MgCl_2_ | 25mM | 4µl |
| DTT | 0.1M | 2µl |
| dNTPs | 10mM | 1µl |
| T4gene32 protein (Roche) |  | 1µl |
| Superscript III (Invitrogen) |  | 1µl |

1. Incubate at 50°C for 90min, 85°C for 5min, then add 1µl RNase H (2U) and incubated at 37°C for 20min.
2. Purify the cDNA using the RNAeasy mini-elute kit (Qiagen) and elute in a volume of 12µl with RNAse free water.

**D. Amplification of cDNA**

1. Second strand cDNA synthesis: Set up the reaction mix tabulated below and place the tubes in thermo-cycler programed with the conditions; 98°C for 20sec, 95°C for 1min, 50°C for 60min and 72°C for 60min.

| Phusion buffer (Finnzymes) | 5X | 10µl |
| --- | --- | --- |
| SL-4 primer | 0.05pM | 1µl |
| dNTPs | 10mM | 1µl |
| Phusion Pol (Finnzymes) |  | 1µl |
| cDNA mix |  | 12µl |
| Nuclease free water |  | 25µl |

*SL-4 (5’GATCTACAGTTTCTGTACTAT3’)*

1. Amplification of the cDNA: To the second strand cDNA synthesis mix, add 1µl of each, SL20 (5pM, 5’ACAGTTTCTGTACTATATTG3’), T3 (5pM), and Phusion polymerase. Run the PCR for 10 cycles at 95°C for 1min, 60°C for 3min and 72°C for 5min with a final extension at 72°C for 10min.
2. Purification of cDNA using the QIAquick PCR purification kit (Qiagen) eluting in 30µl of EB buffer. Quantify the DNA using the Qubit 2.0 fluorometer (Invitrogen).
