# Supplementary material for: Methods to Determine the Transcriptomes of Trypanosomes in Mixtures with Mammalian Cells: The Effects of Parasite Purification and Selective cDNA Amplification
Source: PLoS Negl Trop Dis. 2014 Apr 17;8(4):e2806. doi: 10.1371/journal.pntd.0002806 (PMC3990519; doi:10.1371/journal.pntd.0002806)
Supplement: Table S2 — Calculations of expected RNA amounts in lymphocytes and trypanosomes, and amounts needed for sequencing: details and sources of the information. (DOCX) [file pntd.0002806.s007.docx]

**Supplementary Table S2**

|  |  | ref |
| --- | --- | --- |
| Number of lymphocytes/ml blood | 1-4 x 10^6^ | 1 |
| Number of lymphocytes / ml CSF (stage II minimum) | 5000 | 2 |
| Number of lymphocytes / ml CSF (stage II *T. rhodesiense*, Tanzania) | 1.3 x 10^5^ | 3 |
| Number of lymphocytes / ml CSF (stage II *T. rhodesiense*, Uganda) | 2 x 10^4^ | 3 |
| *T. gambiense* blood parasites/ml (maximum) | ~10^4^ | 4 |
| *T. rhodesiense* blood or CSF parasites/ml (Uganda, maximum) | ~10^6^ | 5 |
| *T. rhodesiense* in stage II CSF sample, total (Uganda, median) | ~2 x 10^4^ | 5 |
| Total RNA content of a trypanosome | 0.5 pg | 6. |
| Expected yield of poly(A)+ RNA from 10^6^ trypanosomes (maximum) | 40 ng | 5, 6 |
| Yield of total RNA from 10^6^ lymphocytes (average) | 2 µg | 7 |
| Expected yield of total poly(A)+ RNA from 10^6^ lymphocytes | 200 ng | 5 |
| Number of mRNAs per trypanosome | 2 x 10^4^ | 6 |
| Proportion of each mRNA that is coding region | 50% | 8. |
| Number of mRNAs per cell from a coding region (modal value) | 1-2 | 9 |
| % of total mapped trypanosome reads for an mRNA present at 2 copies per cell | 0.01% | 9 |

1. Wickipedia

2. Diagnostic threshold (WHO guidelines).

3. Kuepfer I, Hhary EP, Allan M, Edielu A, Burri C, et al. (2011) Clinical presentation of *T. b. rhodesiense* sleeping sickness in second stage patients from Tanzania and Uganda. PLoS Negl Trop Dis 5: e968.

4. Blum J, Neumayr A, Hatz C (2011) Human African trypanosomiasis in endemic populations and travellers. Eur J Clin Microbiol Infect Dis.

5. Our unpublished observations. Numbers vary enormously for both parasites and lymphocytes. Importantly, the trypanosome:lymphocyte ratio is usually below 1:10 in both blood and CSF. We assume 10% poly(A)+ RNA in a total RNA sample.

6. Haanstra J, Stewart M, Luu V-D, van Tuijl A, Westerhoff H, et al. (2008) Control and regulation of gene expression: quantitative analysis of the expression of phosphoglycerate kinase in bloodstream form Trypanosoma brucei. J Biol Chem 283: 2495-2507.

7. Eikmans M, Rekers NV, Anholts JD, Heidt S, Claas FH (2013) Blood cell mRNAs and microRNAs: optimized protocols for extraction and preservation. Blood 121: e81-89.

8. Based on the fact that most of the chromosome (outside telomeric regions) is transcribed and toughly 50% is coding. Genome ref. Since most mRNAs have several different polyadenylation sites and the 3’-UTRs are rather repetitive and AT-rich, the current practice, when assigning reads to genes, is to use the counts from coding regions only.

9. Manful T, Fadda A, Clayton C (2011) The role of the 5'-3' exoribonuclease XRNA in transcriptome-wide mRNA degradation. RNA 17: 2039-2047.
